# The synthesis, degradation and biological function of trehalose- 6-phosphate

**DOI:** 10.1007/s44154-025-00235-8

**Published:** 2025-05-30

**Authors:** Yangzhi Liu, Boqiang Li, Tong Chen, Shiping Tian, Zhanquan Zhang

**Affiliations:** 1 Key Laboratory of Plant Resources, Institute of Botany, Chinese Academy of Sciences, Beijing, 100093 China; 2 State Key Laboratory of Vegetable Biobreeding, Institute of Vegetables and Flowers, Chinese Academy of Agricultural Sciences, Beijing, 100081 China; 3 University of Chinese Academy of Sciences, Beijing, 101499 China

**Keywords:** Trehalose- 6-phosphate, Metabolism pathway, Regulatory function, Virulence, Development

## Abstract

Trehalose-6-phosphate (T6P), an intermediate in trehalose metabolic pathways, is ubiquitously present in nearly all cellular organisms except vertebrates. The most well-characterized metabolic route involves its synthesis by trehalose-6-phosphate synthase (TPS) and dephosphorylation to trehalose by trehalose-6-phosphate phosphatase (TPP) in the TPS/TPP pathway. Besides, alternative trehalose metabolic pathways aslo exist. In addition to being the precursor of trehalose synthesis, T6P functions as a signal molecule regulating various biological processes. In plants, T6P inhibits SnRK1 (Sucrose-nonfermenting 1 Related Kinase 1), while in fungi, T6P primarily inhibits hexokinase and regulates glycolysis. Notably, TPS and TPP themselves also have some regulatory functions. Genetic studies reveal that deletion of *TPS* or *TPP* usually causes developmental and virulence defects in fungi, bacteria and invertebrates. Given that TPS and TPP have important biological functions in pathogenic fungi but are absent in humans and vertebrates, they are ideal targets for fungicide development. This review summarizes trehalose metabolic pathways and the multifaceted roles of T6P in plants, fungi and invertebrates, providing a comprehensive overview of its biological functions. Additionally, it discusses some reported TPS/TPP inhibitor to offer insights for pathogen control strategies.

## Introduction

There are many kinds of small molecular compounds in organisms. They play important roles in homeostasis, metabolism and signal transduction. Trehalose- 6-phosphate (T6P) is the metabolic intermediate in trehalose biosynthesis, in which glucose- 6-phosphate (G6P) and UDP-glucose are catalyzed into T6P and then T6P dephosphorylates into trehalose (Avonce et al. 2006). Interestingly, trehalose synthesis pathways are found in all cellular organisms except vertebrates (Richards et al. 2002). Unlike trehalose that protects cells from diversity of external stresses and supplies energy when necessary (Eleutherio et al. 1993; Hottiger et al. 1994; Hounsa et al. 1998; Lewis et al. 1995), T6P is considered to be a signal molecule in regulating various important life processes (Fichtner and Lunn 2021).

In plants, T6P inhibits SnRK1 (Sucrose-nonfermenting 1 Related Kinase 1), an important energy sensor (Zhai et al. 2018; Zhang et al. 2009). T6P, SnRK1 and transcriptional factor NAC23 form a positive feedback regulatory pathway to sense sucrose and consequently regulate the energy balance and circadian rhythm (Li et al. 2022). Meanwhile, T6P also participates in starch accumulation, development, branching and flowering, and the content change of T6P can alter diverse stress resistances of plants (Fichtner and Lunn 2021). In fungi, T6P mainly inhibits hexokinases and regulates glucose metabolism (Blázquez et al. 1993), as well as disrupting development and virulence (Chen et al. 2021; Foster et al. 2003). As for bacteria and invertebrates, T6P is also related to virulence (Kormish and McGhee 2005; Korte et al. 2016; Wang et al. 2022; Xiong et al. 2016). Moreover, TPS and TPP themselves also have regulatory functions in some important life processes, such as autophagy (Kim et al. 2021). Therefore, TPS and TPP are very desirable targets for pesticide and drug design.

In this review, we summarize the T6P metabolic pathways and its regulatory functions to help readers to have a complete picture of T6P.

## Synthesis and degradation of T6P

**Fig. 1:**
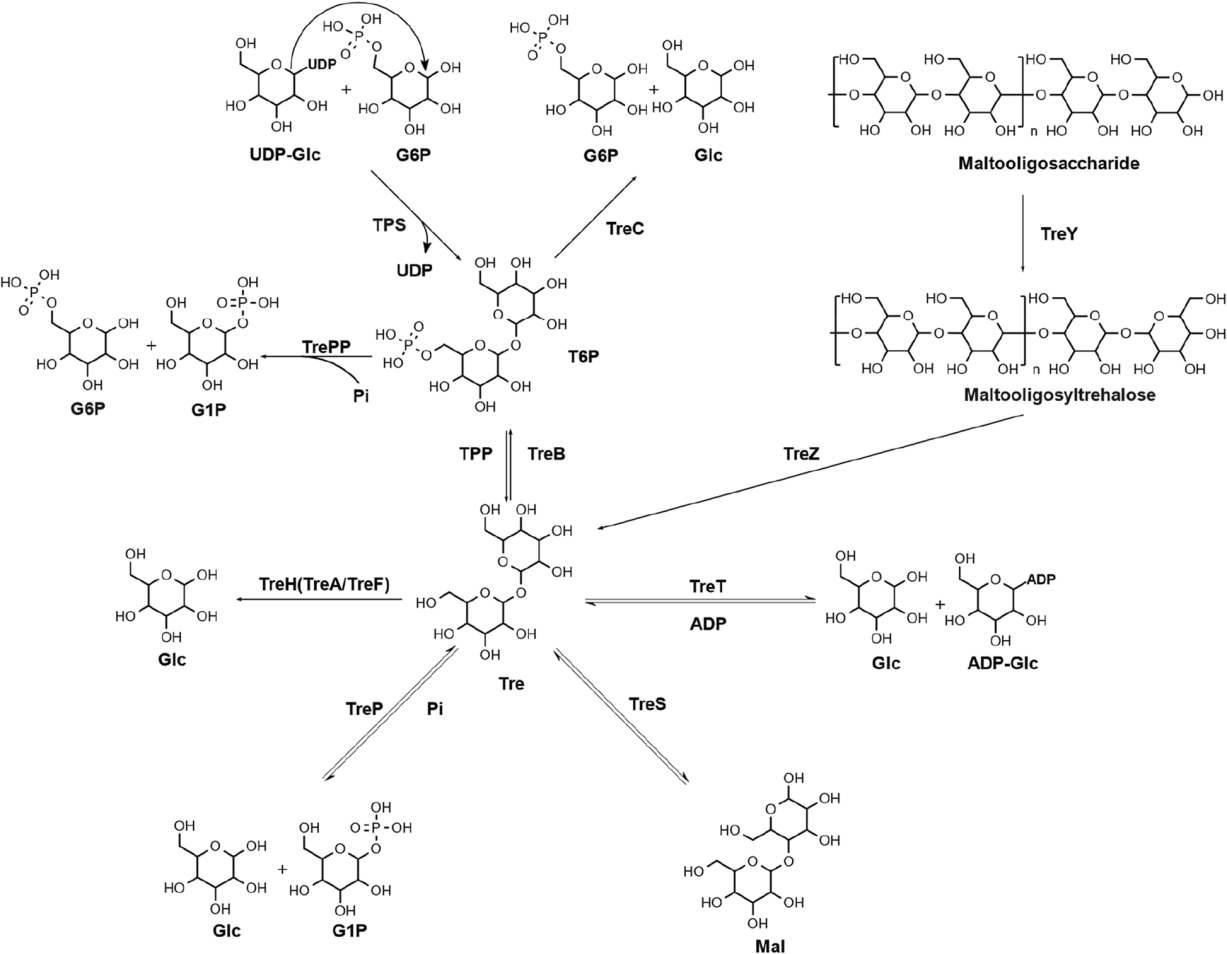
Trehalose and T6P metabolic pathways. Trehalose synthesis pathways: TPS/TPP pathway, UDP-glucose and glucose- 6-phosphate are converted to T6P and UDP by TPS, T6P is converted to trehalose and phosphate group by TPP; TreY/Z pathway, maltooligosaccharide is converted to maltooligosyltrehalose by TreY, trehalose is shed from maltooligosyltrehalose by TreZ. Trehalose degradation pathways: TreB/C pathway, trehalose is converted to T6P by TreB, T6P is converted to glucose- 6-phosphate and glucose by TreC; TreA pathway, trehalose is directly converted to glucose by TreA. Reversible trehalose metabolic pathways: TreP pathway, trehalose and phosphate group are converted to glucose and glucose- 1-phosphate by TreP; TreS pathway, trehalose is converted to maltose by TreS; TreT pathway, trehalose and ADP are converted to glucose and ADP-glucose by TreT. T6P degradation pathway: TrePP pathway, T6P and phosphate group are converted to glucose- 6-phosphate and glucose- 1-phosphate by TrePP. UDP-Glc, UDP-glucose; G6P, glucose- 6-phosphate; Glc, glucose; G1P, glucose- 1-phosphate; Pi, phosphate group; T6P, trehalose- 6-phosphate; Tre, trehalose; ADP-Glc, ADP-glucose; Mal, maltose

The synthesis and degradation of T6P are closely linked with trehalose, a disaccharide composed of two D-glucose molecules connected by an (α,α− 1,1) glycosidic linkage. Therefore, in order to fully understand the metabolic pathways of T6P, a detailed explanation of the trehalose metabolic pathway must be introduced (Fig. 1).

The primary pathway for trehalose synthesis is the TPS/TPP pathway. This pathway is present in a wide range of organisms including bacteria, archaea, metazoa, fungi, and plants. In this pathway, trehalose- 6-phosphate synthetase (TPS) catalyzes the synthesis of T6P from G6P and UDP-glucose, which then dephosphorylates into trehalose with the help of trehalose- 6-phosphate phosphatase (TPP) (Avonce et al. 2006). In *Escherichia coli*, there is a single TPS enzyme (OtsA) and a single TPP enzyme (OtsB), whereas eukaryotes possess multiple TPSs and TPPs. For instance, *Saccharomyces cerevisiae* has four proteins with TPS or TPP domains: ScTPS1, ScTPS2, ScTPS3, and ScTSL1. Among these, ScTPS1 only has TPS domain and others have both TPS and TPP domains. The four proteins form a holoenzyme complex to mediate trehalose synthesis. ScTPS1 functions as a synthetase, ScTPS2 as a phosphatase, and ScTPS3 and ScTSL1 play regulatory roles (Bell et al. 1998). In *Arabidopsis thaliana*, there is greater diversity in TPSs and TPPs, with a total of 11 TPSs and 10 TPPs. The 11 TPSs are further classified into Class I and Class II. Class I TPSs, such as AtTPS1, AtTPS2, AtTPS3, and AtTPS4, have both TPS and TPP domains. They all have TPS functions but do not have TPP functions (Leyman et al. 2001). Class II TPS proteins, containing AtTPS5 - 11, also possess both TPS and TPP domains (Fichtner and Lunn 2021). Despite this, they do not rescue yeast mutants lacking TPS1 or TPS2, indicating a regulatory rather than metabolic role of Class II TPSs (Ramon et al. 2009; Vandesteene et al. 2010; Vogel et al. 2001). In fact, phylogenetic analysis shows the structure of Class II TPSs in *A. thaliana* are more similar to regulatory subunits ScTPS3 and ScTSL1 in *S. cerevisiae* (Lunn 2007). Besides, the interaction between Class I OsTPS1 and Class II OsTPS8 in *Oryza sativa* is verified by coimmunoprecipitation assays and bimolecular fluorescence complementation (Zang et al. 2011). The 10 TPPs are AtTPPA-J, they are all able to complement Δ*Sctps2* mutants of *S. cerevisiae* but have different locations in the cell (Fichtner and Lunn 2021). Although there are variety of TPSs and TPPs in different organisms, the TPS domain and TPP domain they contain are highly conserved in catalytically relevant residues (Avonce et al. 2006).

Besides the TPS/TPP pathway, certain bacteria and archaea also possess a TreY/Z pathway (Kato et al. 1996; Maruta et al. 1996a, b). This pathway involves the conversion of terminal (α,α− 1,4) glycosidic bonds of maltooligosaccharides, starch, or glycogen into (α,α− 1,1) glycosidic bonds, resulting in the production of maltooligosyltrehalose catalyzed by maltooligosyltrehalose synthase (TreY). Subsequently, maltooligosyltrehalose trehalohydrolase (TreZ) catalyzes the dissociation of trehalose from maltooligosyltrehalose. The TreY and TreZ enzymes found in the thermophilic archaeon *Sulfolobus solfataricus* KM1 exhibit high thermostability and have been utilized in the industrial production of trehalose from starch (Kato et al. 1996).

Meanwhile, trehalose can be degraded by the TreH and TreB/C pathways. The TreB/C pathway is mainly found in bacteria. In *E. coli*, TreB/C pathway is involved in both T6P synthesis and degradation. In low osmolarity conditions, extracellular trehalose is transported into cells and converted to T6P by the trehalose-specific enzyme II (TreB) of the phosphotransferase system (PTS). Subsequently, the T6P is hydrolyzed to glucose and G6P by trehalose- 6-phosphate hydrolase (TreC) (Klein et al. 1995; Vanaporn and Titball 2020). The expression of *treB* and *treC* is repressed by the repressor TreR, and T6P can bind to TreR to relieve this repression (Horlacher and Boos 1997). Unlike TreB/C pathway, TreH pathway broadly exists in bacteria, fungi, plants and animals. In *E. coli*, there are two isoforms of TreH, named TreA and TreF. TreA is located in the periplasmic space and directly hydrolyzes trehalose to glucose under high osmolarity conditions (Uhland et al. 2000). The glucose produced is then taken up by the PTS (Horlacher et al. 1996). TreF is located in the cytoplasmic compartment and degrades trehalose synthesized under high osmolarity within the cells (Uhland et al. 2000). In *S. cerevisiae*, TreH is divided into neutral trehalase and acid trehalase. Neutral trehalases are Nth1 and Nth2, they are located in cytoplasm and utilize intracellular trehalose under neutral pH conditions (Parrou et al. 1999; Sakaguchi 2020). Ath 1 is the acid trehalase located in vacuoles, and catalyzes extracellular trehalose at low pH conditions (Nwaka and Holzer 1998). In *A. thaliana* only one TreH named AtTRE1 is reported. AtTRE1 is a plasma membrane-bound enzyme and responsible for extracellular trehalose degradation (Frison et al. 2007). Although there is no trehalose in vertebrates, TreH exists in vertebrates to help utilize trehalose, such as TREH in *Homo sapiens* (Ishihara et al. 1997).

In addition to the irreversible pathways mentioned above, there are also reversible pathways known as TreP, TreS, and TreT pathways. TreP converts trehalose and phosphate groups into glucose- 1-phosphate and glucose (Wannet et al. 1998). TreS converts maltose into trehalose, utilizing a similar reaction mechanism as TreY, which involves converting the (α, α− 1, 4) glycosidic bond of maltose into (α, α− 1, 1) glycosidic bond (Vanaporn and Titball 2020). TreT catalyzes the conversion of trehalose and ADP into ADP-glucose and glucose (Qu et al. 2004). These three pathways are all present in bacteria.

Finally, there is a TrePP pathway that does not refer to trehalose. In gram-positive bacterium and fungi, a T6P can also gather a phosphate group to generate a G6P and a glucose- 1-phosphate catalyzed by trehalose- 6-phosphate phosphorylase (TrePP) (Andersson et al. 2001; Eis and Nidetzky 1999). The glucose- 1-phosphate can subsequently convert to G6P by phosphoglucomutase.

## T6P functions in plants

### Multiple functions of T6P

**Fig. 2:**
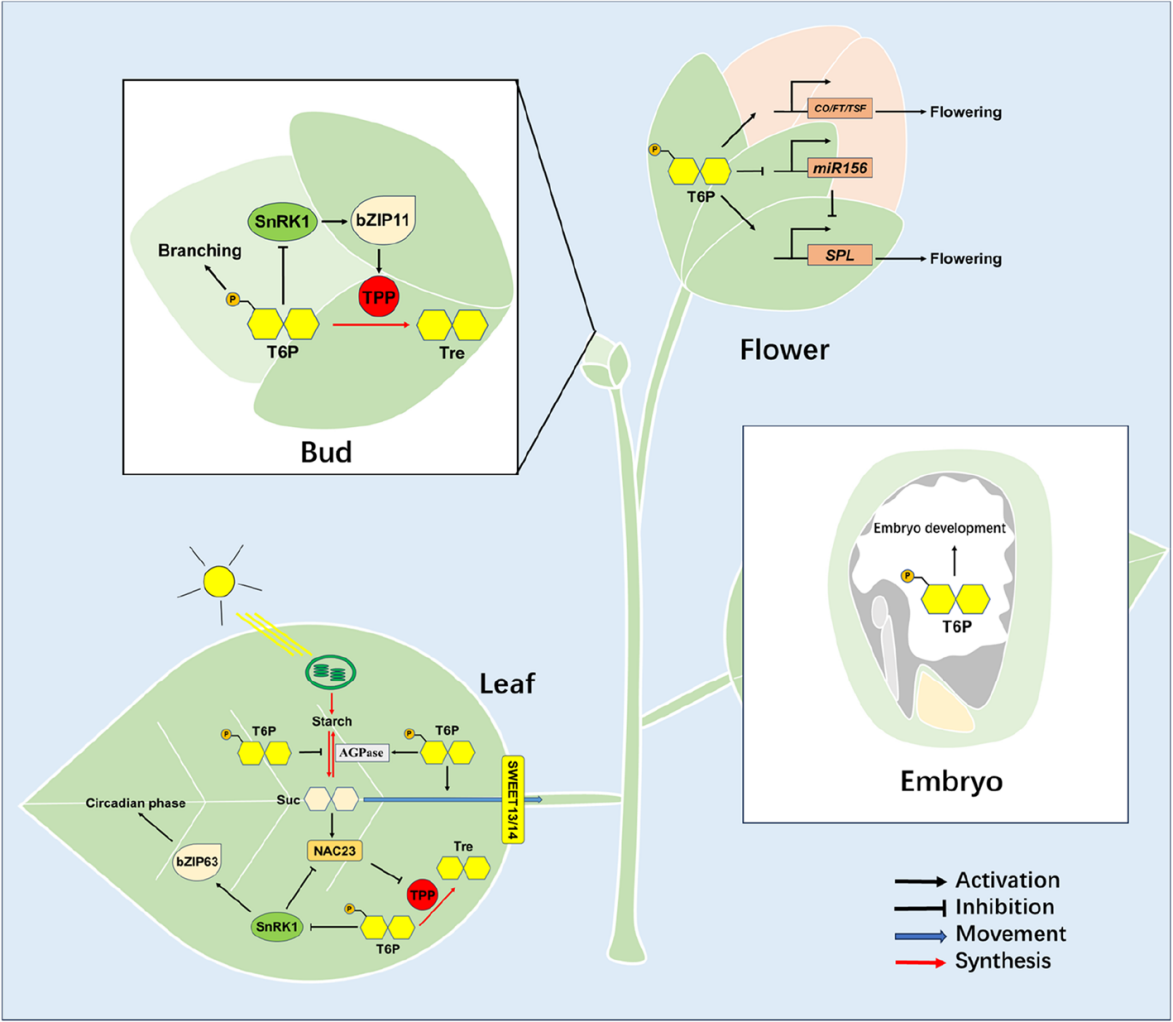
A model of T6P regulatory pattern in plants. In embryos, T6P can promote embryo development. In buds, T6P can promote branching, and maintain its concentration by inhibiting SnRK1 to decrease the activity of transcription factor bZIP11, which promotes the expression of *tpp*. In leaves, sucrose activates transcription factor NAC23 which inhibits the expression of *tpp*, leading to the accumulation of T6P. T6P then inhibits SnRK1, thus relieves the inhibition of SnRK1 on NAC23, forming a positive feedback to increase its concentration. Meanwhile, T6P decrease the sucrose level by inhibiting starch degradation, activating AGPase and promoting sucrose transport to sink organs. In addition, T6P can inhibit bZIP63 by SnRK1 to regulate circadian phase. In flowers, T6P promotes flowering by increasing the expression of florigenic proteins *CO*, *FT* and *TSF*, and transcription factor *SPL*, as well as decreasing the expression of *miR156*. T6P, trehalose- 6-phosphate; Suc, sucrose

T6P is recognized as a signaling molecule that regulates various physiological activities in plants, such as starch accumulation, development, branching, and flowering (Fig. 2).

In 2005, Kolbe observed that overexpressing *otsA* of *E. coli* in *Arabidopsis thaliana*, ADP-glucose pyrophosphorylase is activated, leading to an increase in starch synthesis. Conversely, overexpressing *otsB* of *E. coli* in *A. thaliana*, the opposite effect is observed, suggesting that T6P can activate ADP-glucose pyrophosphorylase and enhance starch synthesis (Kolbe et al. 2005). Furthermore, subsequent studies have shown that T6P can inhibit starch degradation both during the day and night (Ishihara et al. 2022; Martins et al. 2013), as well as reduce sucrose level (Figueroa et al. 2016).

The *A. thaliana* Δ*tps1* mutants were initially thought to be recessive embryo lethal due to growth arrest at an early stage (Eastmond et al. 2002). To study the phenotypic change caused by *tps1* deletion in *A. thaliana*, Dijken utilized the dexamethasone induced expression system to introduce *tps1* into *A. thaliana* Δ*tps1* mutants and ceased dexamethasone supply after germination, which eliminated the embryo lethality. They subsequently found that *tps1* deletion led to a reduction in the root meristematic region, inhibited leaf growth, and prevented floral transition (van Dijken et al. 2004). Subsequently, in 2006, Gómez observed that wrinkled Δ*tps1* seeds of *A. thaliana* were not entirely lethal. When these wrinkled seeds underwent cold stratification and were incubated on agar medium for 1–3 weeks, approximately 30–40% of the embryos expanded and emerged from the seed coat (Gómez et al. 2006). However, these seeds exhibited slow growth and remained in the vegetative stage until senescence. In pea, T6P was found to induce the expression of the auxin biosynthesis gene *TAR2* (*Tryptophan Aminotransferase Related2*), leading to an increase in the 4-Cl-IAA (4-Cl-indoleacetic acid) level during the later stages of embryo development (Meitzel et al. 2021). Interestingly, T6P was also found to accumulate in senescent leaves alongside sugars, highlighting its importance in plant development (Wingler et al. 2012).

In addition to embryo development, T6P also participates in branching of plants. In *A. thaliana*, TPS1 is predominantly synthesized in axillary buds and the subtending vasculature, as well as in the leaves and stem vasculature. Increase of T6P level in axillary buds and vasculature promotes branching and decrease of T6P level inhibits branching (Fichtner et al. 2021). In decapitated garden pea, T6P accumulates in axillary buds releasing from dormancy (Fichtner et al. 2017). In maize, the expression of *RA1*, a transcriptional regulator containing a C2H2 zinc finger, is down-regulated in *RA3* (*tpp*) knock-down mutants, leading to the production of more and longer axillary meristems. This suggests that RA3 or T6P may play a role in regulating inflorescence architecture in maize (Satoh-Nagasawa et al. 2006).

Flowering is a crucial stage in the life cycle of plants, and the presence of T6P plays a critical role in this process. Within the leaves, T6P triggers the activation of key florigenic proteins expression such as *CO* (*CONSTANS*), *FT* (*FLOWERING LOCUS T*), and *TSF* (*TWEEN SISTER OF FT*), along with sucrose transporters SWEET13 and SWEET14, in response to sucrose accumulation (Fichtner et al. 2021; Wahl et al. 2013). Essentially, an adequate amount of sucrose signifies sufficient energy for the flowering. Moreover, in shoot apical meristems, T6P regulates the expression of flowering-time and flower-patterning genes by the age pathway (Wahl et al. 2013). Specifically, T6P suppresses the expression of *miR156*, thereby facilitating the transition from vegetative to reproductive phases in *A. thaliana*, and may directly impacts *SPL (SQUAMOSA PROMOTER BINDING PROTEIN-LIKE)* expression independently of *miR156* regulation (Ponnu et al. 2020; Zhang et al. 2022).

In addition to the mentioned above, T6P is also involved in many other physiological processes as well. Overexpression of bacterial *otsA* in *A. thaliana* leads to significant changes in transcriptional abundance, affecting various biological processes, such as photosynthesis, sucrose export, C/N interactions, ribosome assembly, translation, light-signaling pathways, circadian clock, and hormone signaling pathways (Avidan et al. 2024). High TPS1 activity in *A. thaliana* results in reduced endogenous sucrose level and thermotolerance, while disruption of the T6P signal leads to transitional sucrose accumulation and improves heat resistance (Reichelt et al. 2023). Additionally, overexpression of wheat *tps11* in *A. thaliana* enhances cold tolerance, highlighting the positive correlation between T6P level and cold resistance, and the negative correlation with heat resistance (Liu et al. 2019). Studies by Harthill in 2006 revealed that phosphorylated forms of TPS5, TPS6, and TPS7 in *A. thaliana* can interact with 14-3-3 proteins (phospho-binding proteins that regulate multiple signaling pathways), with N-terminal phosphorylation of TPS5 being essential for this interaction, hinting at a potential downstream role in SnRK1 signaling (Harthill et al. 2006). In *Hevea brasiliensis*, *Hbtps5* expression is downregulated after tapping, an agronomic manipulation that stimulates latex production, indicating its regulatory function (Zhou et al. 2022). Drought stress decreases *Grapevine tps1* expression, emphasizing the importance of T6P level in drought resistance (Morabito et al. 2021). Furthermore, TPP can also influence T6P level and downstream processes. Overexpression of *AttppF* in *A. thaliana* improves drought tolerance (Lin et al. 2019). CqTPP and Class II CqTPSs in *Chenopodium quinoa* participate in the response to saline-alkali stress (Wang et al. 2023).

Given the wide-ranging physiological activities of T6P, TPS or TPP can be used as a target for crop yield and stress resistance improvement.

### Sugar-T6P-SnRK1 nexus, the T6P level regulation system

The importance of T6P has led to decades of research on its regulation mechanism. In 1998, the *tps* and *tpp* genes in *A. thaliana* were first identified, and their functions were confirmed through complementation in yeast Δ*tps1* or Δ*tps2* mutants (Blázquez et al. 1998; Vogel et al. 1998). Subsequently, in 2002, Eastmond discovered that the Δ*tps1* mutants in *A. thaliana* were recessive embryo lethal, with embryo development halting at the cell expansion stage when the sucrose level increased. High exogenous sucrose level further inhibited the development of Δ*tps1* mutants embryos, contrasting with the promotion observed in wild type plants. These findings established a connection between TPS and sucrose in plants. Interestingly, exogenous trehalose was unable to rescue the Δ*tps1* mutants, indicating that the null phenotype is due to T6P decrease rather than trehalose (Eastmond et al. 2002). Overexpression of *tpp* in *A. thaliana*, which also lacks T6P, led to growth inhibition in the presence of exogenous sucrose. Conversely, sucrose promoted the development of plants overexpressing *tps* and accumulating T6P, suggesting that T6P acts as a crucial signal in response to sucrose level (Schluepmann et al. 2003). Moreover, exogenous sucrose was shown to increase T6P level in *A. thaliana* seeds (Lunn et al. 2006).

In 2004, Schluepmann first discovered the association between T6P level and AtKIN11, a subunit of SnRK1, in *A. thaliana* seedlings. Through q-PCR analysis, it was confirmed that the expression level of *AtKIN11* are positively correlated with T6P level (Schluepmann et al. 2004). Subsequently, in 2009, Zhang utilized *A. thaliana* lines expressing *E. coli otsA* and *otsB* to investigate the inhibitory effect of T6P on SnRK1 activity in all tissues except mature leaves (Zhang et al. 2009). This study highlighted a potential relationship between T6P and SnRK1. Building on this, in 2018, Zhai employed microscale thermophoresis (MST) to demonstrate the interaction between T6P and KIN10, another subunit of SnRK1 (Zhai et al. 2018). The findings indicated that T6P binds to KIN10, reducing its affinity to GRIK (Geminivirus Rep-Interacting Kinase) and subsequently decreasing KIN10 Thr175 phosphorylation. Phosphorylated KIN10 in turn mediates the phosphorylation of WRINKLED1, a key transcriptional activator of fatty acid synthesis (Zhai et al. 2017). The research suggests that KIN10, rather than KIN11, plays a crucial role in T6P signaling. This is supported by the fact that *KIN10* mutation can rescue *A. thaliana* Δ*tps1* mutants, while *KIN11* mutation cannot, underscoring the non-redundant functions of KIN10 and KIN11 (Zacharaki et al. 2022). Overall, sucrose, T6P and SnRK1 are closely linked together.

But how does sucrose affect T6P level? In 2014, Yadav observed that the increase in T6P level following sucrose induction is largely hindered by cycloheximide (a bacterial toxin that interferers with protein biosynthesis), implying the presence of an intermediary protein that connects sucrose to TPS/TPP (Yadav et al. 2014). Building on this, in 2022, Li identified the transcription factor OsNAC23 in rice, which is activated by sugar and directly suppresses the transcription of *tpp*, resulting in elevated T6P level. Furthermore, the enzyme SnRK1, which is inhibited by T6P, facilitates the phosphorylation and degradation of OsNAC23 (Li et al. 2022). In addition to OsNAC23, the transcription factor bZIP11 also appears to influence *tpp* expression level. In contrast to the repressive role of OsNAC23, bZIP11 activates TPP, leading to a reduction in T6P level and shoot branching inhibition. Interestingly, SnRK1 is able to maintain high level of bZIP11 (Hellens et al. 2023). Furthermore, SnRK1 has been found to regulate bZIP63, another transcription factor that controls the circadian oscillator gene *PRR7*. This suggests that T6P can modulate the circadian phase through its interaction with SnRK1 (Frank et al. 2018).

## T6P functions in fungi

**Fig. 3:**
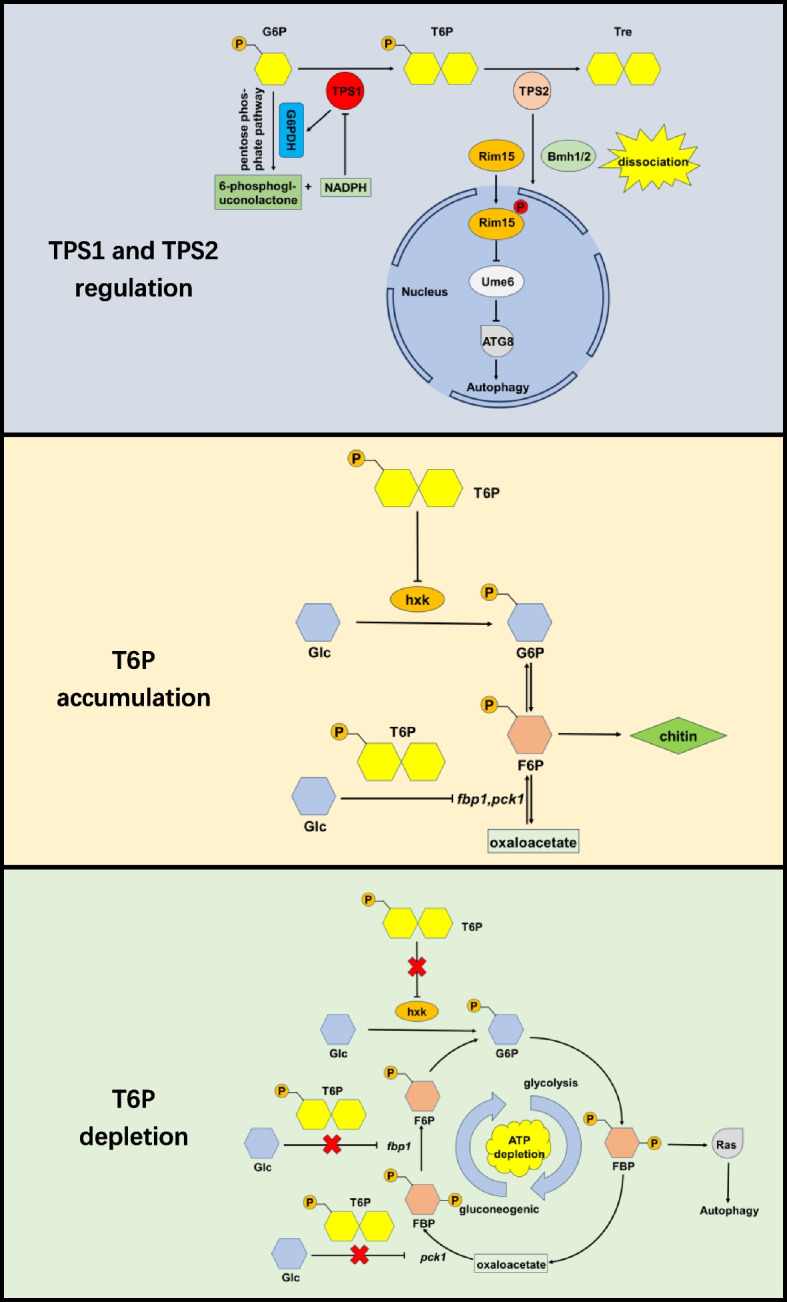
A model of T6P, TPS1 and TPS2 regulatory pattern in fungi. TPS1 can activate G6PDH and promote more glucose to enter pentose phosphate pathway to produce NADPH. NADPH in return inhibits TPS1 to keep T6P balance. TPS2 can dissociates the Rim15/Bmh1/2 complex under nitrogen starvation. Free Rim15 then moves into the nucleus and finally triggers autophagy through Ume6-ATG8. T6P accumulation will inhibit hexokinases activity and the expression of gluconeogenesis related genes *fbp1* and *pck1*, weakening the glycolysis and gluconeogenesis at the same time. It resulted in the decrease of chitin synthesis. T6P decrease relieves the inhibition on hexokinases and *fbp1* and *pck1* expression, leading to excess glycolysis and gluconeogenesis. It causes ATP depletion. Meanwhile, fructose- 1,6-biophosphate produced by glycolysis can trigger autophagy by activate Ras. G6P, glucose- 6-phosphate; T6P, trehalose- 6-phosphate; Tre, trehalose; Glc, glucose; hxk, hexokinase; F6P, fructose- 6-phosphate; FBP, fructose- 1,6-biophosphate

In fungi, in addition to the regulatory function of T6P, TPS and TPP also play important roles in some life processes (Fig. 3).

In *S. cerevisiae*, it has been found that T6P inhibits hexokinases, particularly hexokinase II (Blázquez et al. 1993). Hexokinases play a crucial role in the initial step of glycolysis by catalyzing the phosphorylation of glucose to glucose- 6-phosphate. In Δ*tps1* mutants, the level of T6P decrease, resulting in the relief of inhibition on hexokinases and causing an excessive influx of glucose into glycolysis (Hohmann et al. 1996). This leads to the removal of glucose inhibition on the gluconeogenic genes *pck1* and *fbp1*, thereby enhancing gluconeogenesis (Deroover et al. 2016; Vicente et al. 2018). The increased futile cycle between glycolysis and gluconeogenesis may contribute to ATP depletion in Δ*tps1* mutants (Bonini et al. 2003). Furthermore, downstream glycolysis intermediate fructose- 1,6-biophosphate will hyperaccumulate (Bonini et al. 2003), leading to hyperactivation of Ras, a kind of small GTP binding protein. The activation of Ras ultimately triggers cell apoptosis (Peeters et al. 2017).

Deletion of *tps2* leads to T6P accumulation, contrasting with Δ*tps1* mutants in *S. cerevisiae* (de Virgilio et al. 1993). A decrease in chitin synthesis is a significant consequence observed in Δ*tps2* mutants of various fungi, such as *Aspergillus nidulans* (Borgia et al. 1996), *Magnaporthe oryzae* (Chen et al. 2021), *Aspergillus fumigatus* (Puttikamonkul et al. 2010) and *Fusarium graminearum* (Song et al. 2014), although the exact cause remains unclear. The reduction in chitin content weakens the cell wall, resulting in abnormal swelling and lysis of conidia and hyphae (Borgia et al. 1996). This manifests externally as growth retardation and sporulation defects. High osmotic stress can fully complement the growth disruption of mutants, and N-acetylglucosamine provides slight complementation, suggesting that *tps2* deletion disrupts N-acetylglucosamine synthesis, further impacting chitin synthesis and cell wall stability (Borgia et al. 1996; Puttikamonkul et al. 2010). Moreover, the polar distribution of actin and polarisome scaffolding protein is destroyed in Δ*tps2* mutants of *M. oryzae* and *F. graminearum* (Chen et al. 2021; Song et al. 2014). This could also be caused by chitin decrease.

Virulence disruption is observed in both Δ*tps1* and Δ*tps2* mutants of pathogenic fungi. In many fungi, for example, *Candida albicans* (van Dijck et al. 2002), *A. fumigatus* (Puttikamonkul et al. 2010), *M. oryzae* (Chen et al. 2021), *F. graminearum* (Song et al. 2014), *Beauveria bassiana* (Qiu et al. 2020) and so on, Δ*tps2* mutants showed virulence defects. However, *tps1* deletion is also found to disrupt virulence only in *C. albicans* (Zaragoza et al. 1998) and *M. oryzae* (Foster et al. 2003). Moreover, some fungi, like *Aspergillus niger* (Wolschek and Kubicek 1997) and *Botrytis cinerea* (Doehlemann et al. 2006), there are no significant differences between the virulence of Δ*tps1* mutants and wild type strains under normal conditions. This suggests that the decrease in virulence in Δ*tps2* mutants is likely due to excess T6P, while the decrease in virulence in Δ*tps1* mutants may be attributed to a reduction in trehalose rather than the absence of T6P.

Heat shock is observed in both Δ*tps1* and Δ*tps2* mutants across various species. For Δ*tps1* mutants, the exponential cell viability of *S. cerevisiae* greatly reduces at 42 ℃ (Argüelles 1994), *C. albicans* cannot grow on glucose or fructose at 42 ℃ (Zaragoza et al. 1998), and *B. cinerea* has vegetative growth defects and conidia viability decrease at 30 ℃ (Doehlemann et al. 2006). For Δ*tps2* mutants, fermentation of glucose is weakened in *S. cerevisiae* at 42 ℃ (Blázquez et al. 1993), *C. albicans* cells rapidly die at 44 ℃ (van Dijck et al. 2002), the conidia and hyphae of *A. nidulans* swell and lyse at 42 ℃ (Borgia et al. 1996), and *A. fumigatus* growth rate significantly decreases at 45 ℃ (Puttikamonkul et al. 2010). Interestingly, T6P levels show opposite trends between Δ*tps1* and Δ*tps2* mutants, while trehalose contents decrease in both. This suggests that the disruption of trehalose rather than T6P synthesis is likely the main contributing factor of heat shock. Previous studies have demonstrated that trehalose plays a role in stabilizing proteins, thereby enhancing the thermotolerance of yeast (Hottiger et al. 1994).

In *S. cerevisiae* strains expressing *Schizosaccharomyces pombe* hexokinase, which is insensitive to T6P, deletion of *tps1* also leads to significant growth defects on glucose (Bonini et al. 2003). This suggests that T6P is not the sole regulator of glucose metabolism, as TPS1 can also play a crucial role. In fact, TPS1 and TPS2 directly regulate certain downstream proteins. For instance, in *Magnaporthe grisea*, TPS1 can detect G6P, its substrate for T6P synthesis, and activate G6PDH (glucose- 6-phosphate dehydrogenase), a key enzyme in the pentose phosphate pathway that converts G6P to 6-phosphogluconolactone while reducing NADP to NADPH (Wilson et al. 2007). The generated NADPH can bind to TPS1, inhibiting its activity and establishing a negative feedback loop to keep the TPS1 activity at an appropriate level. Moreover, NADP consumption inactivates the Nmr, a class of transcriptional corepressors containing Nmr1, Nmr2, and Nmr3, resulting in the derepression of various downstream transcription factors involved in virulence, secondary metabolism and so on (Wilson et al. 2010). Essentially, *tps1* deletion promotes the inhibition of Nmr on certain transcription factors, leading to a decrease in pathogenicity. In nitrogen-starved *S. cerevisiae*, TPS2 triggers the dissociation of Rim15, a kinase that negatively regulates Ume6, and Bmh1/2, a 14-3-3 protein. Rim15 then translocates into the nucleus and is activated by autophosphorylation. Activated Rim15 inhibits Ume6 by phosphorylation, reliving the inhibition of autophagy-related protein ATG8 and upregulating autophagy (Kim et al. 2021).

## T6P functions in bacteria and invertebrates

Besides plants and fungi, T6P is also found in bacteria and invertebrates. Both Δ*otsA* and Δ*otsB* mutants of *E. coli* are sensitive to high osmotic stress (Giaever et al. 1988). Like the Δ*tps2* mutants in fungi, T6P accumulates in Δ*otsB* mutants of some bacteria, such as *Mycobacterium tuberculosis* (Korte et al. 2016) and *Acinetobacter baumannii* (Hubloher et al. 2020), and decreases their virulence. In model organism *Caenorhabditis elegans*, *gob- 1* (trehalose- 6-phosphate phosphatase) deletion is lethal in early larval, while further deletion of *tps- 1* and *tps- 2* (two trehalose- 6-phosphate synthases) can restore the lethal phenotype. This phenomenon shows that T6P accumulation rather than trehalose deficiency is the main reason for lethality (Kormish and McGhee 2005). Related studies are also carried out in *Brugia malayi*, *tpp* silencing results in serious embryonic development arrest and virulence decrease (Kushwaha et al. 2012).

Especially, trehalose is the major blood sugar in insects (Tang et al. 2018). Therefore, T6P is very important as the intermediate in trehalose biosynthesis. In *Bactrocera minax*, *tpp* silencing can also increase mortality and malformation rate (Wang et al. 2022). Meanwhile, *tps* is also focused on as a control target. It is confirmed that *tps* silencing can result in down-regulation of chitin synthesis related genes in *B. minax* (Xiong et al. 2016), *Bemisia tabaci* (Gong et al. 2022), *Tribolium castaneum* (Chen et al. 2018), *Acyrthosiphon pisum* (Wang et al. 2021), *Heortia vitessoides* (Chen et al. 2020) and *Nilaparvata lugens* (Chen et al. 2010). Among these, except *A. pisum*, abnormal development and lethality are observed after *tps* silencing. However, there is no direct evidence that these abnormal phenotypes are caused by T6P, trehalose or TPS regulatory function disruption may also be the reason.

## Inhibitors of TPS and TPP

It can be seen that TPS and TPP are indeed ideal targets to control fungi, bacteria and insects, because of their importance to development and pathogenicity, as well as their absence in human genome. Hence, looking for effective inhibitor to TPS and TPP proteins is a promising research direction and some excellent progress has been made (Table 1).

**Table 1:** Reported inhibitors of fungi and insects TPS1 and TPS2

Chemical name	Structure	Target	Reference
Lead- 25		MoTPS1	(Xue et al. 2014)
chaetoviridin-A		MoTPS1	(Khan et al. 2022)
GKK1032 A2		MoTPS1	
camptothecin		MoTPS1	
rocaglaol		MoTPS1	
azoxystrobin		MoTPS1	
strobilurin		MoTPS1	
A1 - 4		MoTPS1, MoTPS2	(Chen et al. 2024)
2,6-diamino- 3,5-dicyano- 4-(3,4-dichlorophenyl)− 4H-thiopyran		DmTPS, CfTPS	(Kern et al. 2012)

MoTPS1 is the trehalose- 6-phosphate synthase in *Magnaporthe oryzae*, MoTPS2 is the trehalose- 6-phosphate phosphatase in *Magnaporthe oryzae*, DmTPS is the trehalose- 6-phosphate synthase in *Drosophila melanogaster* and CfTPS is the trehalose- 6-phosphate synthase in* Ctenocephalides felis*

Virtual screening is a commonly used method for protein interacting molecule seeking, using algorithms to simulate combining capacity of proteins to ligands in silico (Sadybekov and Katritch 2023). By screening over 400,000 chemicals, structural optimization of the best candidates and molecular dynamics simulation, a chemical named Lead- 25 is designed. Lead- 25 has excellent affinity to TPS in *M. oryzae* (MoTPS1) about − 13.8 kcal/mol and good water solubility (Xue et al. 2014). Another research screens 39 natural compounds, which have effective antifungal activity to *M. oryzae *in vitro, by silico molecular docking analysis, and find 6 compounds with nice affinity to MoTPS1. They are chaetoviridin-A, about − 7.3 kcal/mol, GKK1032 A2, about − 10.2 kcal/mol, camptothecin, about − 8.7 kcal/mol, rocaglaol, about − 7.3 kcal/mol, azoxystrobin, about − 7.4 kcal/mol and strobilurin, about − 7.7 kcal/mol (Khan et al. 2022). Moreover, a chemical named A1 - 4 is discovered to inhibit both MoTPS1 and MoTPS2 (TPP) by virtual screening, and the binding affinity are − 7.7 kcal/mol and − 6.8 kcal/mol, respectively. Surface plasmon resonance (SPR) shows that the equilibrium dissociation constants (K_D_) between A1 - 4 and MoTPS1/MoTPS2 are 26.2 μM and 59.8 μM. Meanwhile, A1 - 4 can also inhibit the enzyme activity of MoTPS1 and MoTPS2, *M. oryzae* growth rate and virulence (Chen et al. 2024). High throughput screening (HTS) is an experimental method which screens protein interacting molecules by using microtiter plates with hundreds of wells (Entzeroth et al. 2009). As for insect TPS, 4-substituted 2,6-diamino- 3,5-dicyano- 4H-thiopyran derivatives are found to have potent inhibitory effect on *Ctenocephalides felis* TPS (CfTPS) and *Drosophila melanogaster* TPS (DmTPS) by HTS. In particular, 2,6-diamino- 3,5-dicyano- 4-(3,4-dichlorophenyl)− 4H-thiopyran is the most effective one, its IC50 values of DmTPS and CfTPS are respectively 0.2 μM and 0.5 μM under 1.875 μg/mL and 5.39 μg/mL protein concentration (Kern et al. 2012).

The development of artificial intelligence (AI) brings new opportunities to TPS and TPP inhibitor design. AlphaFold2 can provide more accurate protein structures of TPS and TPP, and AI-based algorithms like equivariant neural networks significantly improve the accuracy of virtual docking. Besides screening, AI makes it possible to de novo design TPS and TPP inhibitors tailored to specific requirements (Zhang et al. 2025).

## Conclusion

T6P is an intermediate of TPS/TPP trehalose synthesis pathway. Additionally, there are some other trehalose metabolic pathways, they are TreY/Z pathway for synthesis, TreA and TreB/C pathways for degradation, TreT, TreP and TreS pathways for both synthesis and degradation. T6P can also be degraded by TrePP pathway. The presence of various trehalose metabolic pathways indicates the importance of trehalose and T6P.

In plants, sucrose induces T6P accumulation by activate the transcription factor NAC23 which can inhibit the expression of *tpp*. And two positive feedbacks amplify this process through SnRK1-NAC23-TPP and SnRK1-bZIP11-TPP. Furthermore, T6P participates in starch synthesis, sucrose transportation, branching, embryo development, circadian rhythm regulation, flowering and so on. In fungi, *tps1* deletion will cause T6P synthesis defect and trehalose decrease. Lack of T6P relieves the inhibition of glucose to gluconeogenesis genes *pck1* and *fbp1*. Trehalose decrease weakens the heat resistance. Besides, TPS1 itself can sense G6P and lead it to pentose phosphate pathway to reduce glycolysis flux, preventing glucose growth defect and autophagy caused by fructose- 1,6-biophosphate accumulation. Overall, both glycolysis and gluconeogenesis increase, this futile cycle results in ATP depletion. On the contrary, *tps2* deletion will cause T6P accumulation and trehalose decrease. Excess T6P will inhibit hexokinase and lead to development defect, virulence decrease, sporulation disruption and cell wall structure abnormality. Trehalose decrease also weakens the heat resistance. Besides, TPS2 itself can directly regulate autophagy by Rim15-Ume6-ATG8 pathway under nitrogen starvation. In bacteria and invertebrates, *tps* and *tpp* can also lead to development and virulence defects. As reported, T6P has different functions in plants and fungi, bacteria, invertebrates. T6P in plants can positively regulate many life processes, while in fungi, bacteria and invertebrates, accumulation of T6P leads to serious defects. T6P inhibits SnRK1 in plants but hexokinase in fungi probably because the homologous proteins have different structures in plants and fungi. It suggests that T6P is a multifunctional small molecule, and it may have many other potential target proteins.

Lead- 25, azoxystrobin, GKK1032 A2, camptothecin, chaetoviridin A, rocaglaol and strobilurin are predicted to be effective on inhibiting MoTPS1 activity. Chemical A1 - 4 is predicted to inhibit both MoTPS1 and MoTPS2 and its antifungal effect is verified. 2,6-diamino- 3,5-dicyano- 4-(3,4-dichlorophenyl)− 4H-thiopyran is an effective inhibitor on DmTPS and CfTPS. However, pesticide design should also consider its potential side effects on plant TPS and TPP. The pathogen targeted TPS/TPP inhibitor should not interact with plant TPS and TPP.

## Data Availability

All data and material generated or analyzed during this study are included in this published article.

## Abbreviations

T6P: Trehalose- 6-phosphate
TPS: Trehalose- 6-phosphate synthase
TPP: Trehalose- 6-phosphate phosphatase
SnRK1: Sucrose-nonfermenting 1 related kinase 1
G6P: Glucose- 6-phosphate
NLS: N-terminal nuclear localization signal
4-Cl-IAA: 4-Cl-indoleacetic acid
CO: CONSTANS
FT: FLOWERING LOCUS T
TSF: TWEEN SISTER OF FT
SPL: SQUAMOSA PROMOTER BINDING PROTEIN-LIKE
GRIK: Geminivirus rep-interacting kinase
G6PDH: Glucose- 6-phosphate dehydrogenase
HTS: High throughput screening
AI: Artificial intelligence
